# Impaired metal perception and regulation of associated human foliate papillae tongue transcriptome in long-COVID-19

**DOI:** 10.1038/s41598-024-66079-w

**Published:** 2024-07-04

**Authors:** Barbara Danzer, Mateo Jukic, Andreas Dunkel, Gaby Andersen, Barbara Lieder, Erika Schaudy, Sarah Stadlmayr, Jory Lietard, Timm Michel, Dietmar Krautwurst, Bernhard Haller, Percy Knolle, Mark Somoza, Paul Lingor, Veronika Somoza

**Affiliations:** 1 School of Life Science, Technical University of Munich, Freising, Germany; 2 Leibniz Institute for Food Systems Biology at the Technical University of Munich, Freising, Germany; 3 Department of Neurology, School of Medicine and Health, Klinikum rechts der Isar, Technical University of Munich, Munich, Germany; 4 Department of Physiological Chemistry, Faculty of Chemistry, University of Vienna, Vienna, Austria; 5 Department of Inorganic Chemistry, Faculty of Chemistry, University of Vienna, Vienna, Austria; 6 Institute of AI and Informatics in Medicine, School of Medicine and Health, Technical University of Munich, Munich, Germany; 7 Institute of Molecular Immunology, School of Medicine and Health, Technical University of Munich, Munich, Germany; 8 Chair of Food Chemistry and Molecular Sensory Science, School of Life Sciences, Technical University of Munich, Freising, Germany; 9 German Center for Neurodegenerative Diseases (DZNE), Munich, Germany; 10 Munich Cluster for Systems Neurology (SyNergy), Munich, Germany; 11 Chair of Nutritional Systems Biology, School of Life Sciences, Technical University of Munich, Freising, Germany; 12 Present Address: Institute of Clinical Nutrition, University of Hohenheim, Stuttgart, Germany

**Keywords:** Viral infection, Molecular medicine

## Abstract

Chemosensory impairment is an outstanding symptom of SARS-CoV-2 infections. We hypothesized that measured sensory impairments are accompanied by transcriptomic changes in the foliate papillae area of the tongue. Hospital personnel with known SARS-CoV-2 immunoglobulin G (IgG) status completed questionnaires on sensory perception (*n* = 158). A subcohort of *n* = 141 participated in forced choice taste tests, and *n* = 43 participants consented to donate tongue swabs of the foliate papillae area for whole transcriptome analysis. The study included four groups of participants differing in IgG levels (≥ 10 AU/mL = IgG^+^; < 10 AU/mL = IgG^-^) and self-reported sensory impairment (SSI^±^). IgG^+^ subjects not detecting metallic taste had higher IgG^+^ levels than IgG^+^ participants detecting iron gluconate (*p* = 0.03). Smell perception was the most impaired biological process in the transcriptome data from IgG^+^/SSI^+^ participants subjected to gene ontology enrichment. IgG^+^/SSI^+^ subjects demonstrated lower expression levels of 166 olfactory receptors (OR) and 9 taste associated receptors (TAS) of which OR1A2, OR2J2, OR1A1, OR5K1 and OR1G1, as well as TAS2R7 are linked to metallic perception. The question raised by this study is whether odorant receptors on the tongue (i) might play a role in metal sensation, and (ii) are potential targets for virus-initiated sensory impairments, which needs to be investigated in future functional studies.

## Introduction

The coronavirus disease 2019 (COVID-19) is caused by the severe acute respiratory syndrome coronavirus-2 (SARS-CoV-2). Like previous coronaviruses, SARS-CoV-2 enters the host cell by interacting with the angiotensin-converting enzyme 2 (ACE2) protein, which serves as a receptor for the spike (S) protein. After binding to the cell, the virus is primed mainly by the cellular serine protease TMPRSS2. This enzyme cleaves the S protein, which enables the fusion of viral and cellular membranes^1,2^. SARS-CoV-2 is highly infectious. Chills, fever, cough, headache, and fatigue are frequent symptoms of the acute disease. Furthermore, the impairment of taste, smell, and chemesthesis are highly associated symptoms^3–5^. In the first wave of the COVID-19 pandemic, sensory dysfunction was reported in 41% of SARS-CoV-2 positive patients with mild disease who did not require hospitalization^6^, while olfactory impairments were reported in up to 86% and gustatory dysfunctions in up to 88% of mild-to-moderately symptomatic patients^4^. In a meta-analysis of ten studies, a prevalence of 53% was reported for olfactory dysfunctions, and a meta-analysis of 9 studies found a prevalence of 44% for gustatory impairments^7^. Even though the prevalence of sensory disorder varied considerably in different populations (5–34%^8–11^) in pre-pandemic studies, increasing concern is raised by the post-acute COVID-19 syndrome also called long COVID. Long COVID describes the long-term complications and the persistence of symptoms after the acute phase of the disease^12^. While the sense of taste and smell is restored completely for the majority of patients, the impairment of chemosensory dysfunctions was found to be persistent in 13–30%^6,13–16^ of SARS-CoV-2 infected individuals. This impairs the quality of life and especially the psychological well-being of affected patients^15^. In a cross-sectional study using a questionnaire, no significant association was found for taste or trigeminal sensations with non-COVID-19 or post-COVID-19 participants with smell distortions^17^. Independently of the cause of these distortions, 45% of all these participants described the presence of a metallic taste in the mouth, 31% described burning nasal passages, and 14% a burning sensation in the mouth in that study^17^. Furthermore, distortions in smell were predominantly reported as unpleasant^17^. Thus, we decided to include the taste sensations of spicy and metallic in our study, predominantly focusing on probable unpleasant taste sensations such as bitter, sour, spicy, and metallic. At the same time, sweet was tested as a likely pleasant comparator. Metallic taste perception can occur through oral chemoreceptors and/or retro-nasal smell^18–20^. Especially ferrous sulfate was shown to have a strong retro-nasal component, possibly due to rapid lipid oxidation in the oral cavity^21^. Metallic sensations are mainly known as unwanted off-flavors, and their perception mechanism is still poorly understood. Only a few taste and olfactory receptors are currently hypothesized to contribute to metallic sensation^20^. The SeCoMRI study, which analyzed a cohort of 4,554 hospital employees during the first wave of the COVID-19 pandemic, clearly showed that loss of smell and taste had the highest positive predictive value for a SARS-CoV-2 infection, and seropositivity was associated with a higher number of reported symptoms^22^. Based on this^22^, we hypothesized that changes in sensory perception after a SARS-CoV-2 infection would be associated with characteristic changes in the foliate papillae transcriptome in IgG seropositive participants. In our approach, SSI^+^ IgG^+^ subjects were the main focus of the four groups of participants, whereas SSI^±^ IgG^-^ and SSI^-^ IgG^+^ individuals were included for comparison. Self-reports in questionnaires were complemented with objective taste tests and whole transcriptome microarray analysis of RNA isolated from the foliate papillae of the tongue.

## Results

### Sensory study

The preceding SeCoMRI study investigated SARS-CoV-2 infections in 4554 health care workers during April 14–May 29, 2020 in Munich, Germany, with a focus on infection risk factors, clinical symptoms, and the seroprevalence of SARS-CoV-2 antibodies^22^. Based on the SeCoMRI study participants were matched in age, sex, and self-reported sensory impairment and contacted a second time. In total, 158 participants were enrolled for primary analysis. SARS-CoV-2 seropositivity was determined by IgG ≥ 10 AU/mL (IgG^+^). A questionnaire was used to determine the prevalence of self-reported sensory impairments at the start of our study (Table 1).

**Table 1:** Demographics of the study population.

	SARS-CoV-2 seronegative IgG^−^ < 10 AU/mL *n* = 79		SARS-CoV-2 seropositive IgG^+^ ≥ 10 AU/mL *n* = 79	
	No self-reported sensory impairment (SSI^−^) *n* = 41	Self-reported sensory impairment (SSI^+^) *n* = 38	No self-reported sensory impairment (SSI^−^) *n* = 41	Self-reported sensory impairment (SSI^+^) *n* = 38
Age				
Median ± SD [min–max]	46 ± 13 [24–68]	37 ± 12 [20–63]	50 ± 13 [25–68]	33 ± 13 [21–62]
Gender				
Female [*n* = 95 (100%)]	25 (26.3%)	24 (25.3%)	25 (26.3%)	21 (22.1%)
Male [*n* = *63* (100%)]	16 (25.4%)	14 (22.2%)	16 (25.4%)	17 (27.0%)
Self-reported sensory deficit				
Persistent loss of smell/taste [*n* = *23* (100%)]	0	6 (26.1%)	0	17 (73.9%)
Recovered sense of smell/taste [*n* = 53 (100%)]	0	32 (60.4%)	0	21 (39.6%)
None [*n* = *82* (100%)]	41 (50.0%)	0	41 (50.0%)	0

Further, participants were asked to self-evaluate the intensity of sensory alterations in eight commonly consumed food groups to understand whether subjective alterations of taste correlate with the SARS-CoV-2-IgG status. The following food groups were selected according to their known key sensory profiles^23^: coffee, tea, chocolate, cheese, meats, vegetables, fruits, and bread. However, no differences were found between IgG^−^ and IgG^+^ subjects (see Supplementary Table S1). Complementing the questionnaires and the IgG titer, participants were subjected to sensory taste tests which included the taste qualities metal, spiciness, sweet, sour, and bitter. A qualitative analysis of taste perception was performed 2 weeks after completing the first questionnaire using standardized taste samples and a forced choice test against water by 141 of the participants (Table 2). 17 participants withdrew for personal reasons.

**Table 2:** Numbers of samples per group and study section.

	SARS-CoV-2 seronegative IgG^−^ < 10 AU/mL [n (%)]		SARS-CoV-2 seropositive IgG^+^ ≥ 10 AU/mL [n (%)]	
	No self-reported sensory impairment (SSI^−^)	Self-reported sensory impairment (SSI^+^)	No self-reported sensory impairment (SSI^−^)	Self-reported sensory impairment (SSI^+^)
Questionnaire *n* = 158 (100%)	38 (24%)	41 (26%)	38 (24%)	41 (26%)
Sensory test *n* = 141 (87%)	77 (55%)		64 (45%)	
	39 (28%)	38 (27%)	33 (23%)	31 (22%)
Tongue swabs included *n* = 43 (27%)	15 (35%)	6 (14%)	13 (30%)	9 (21%)

**Figure 1:**
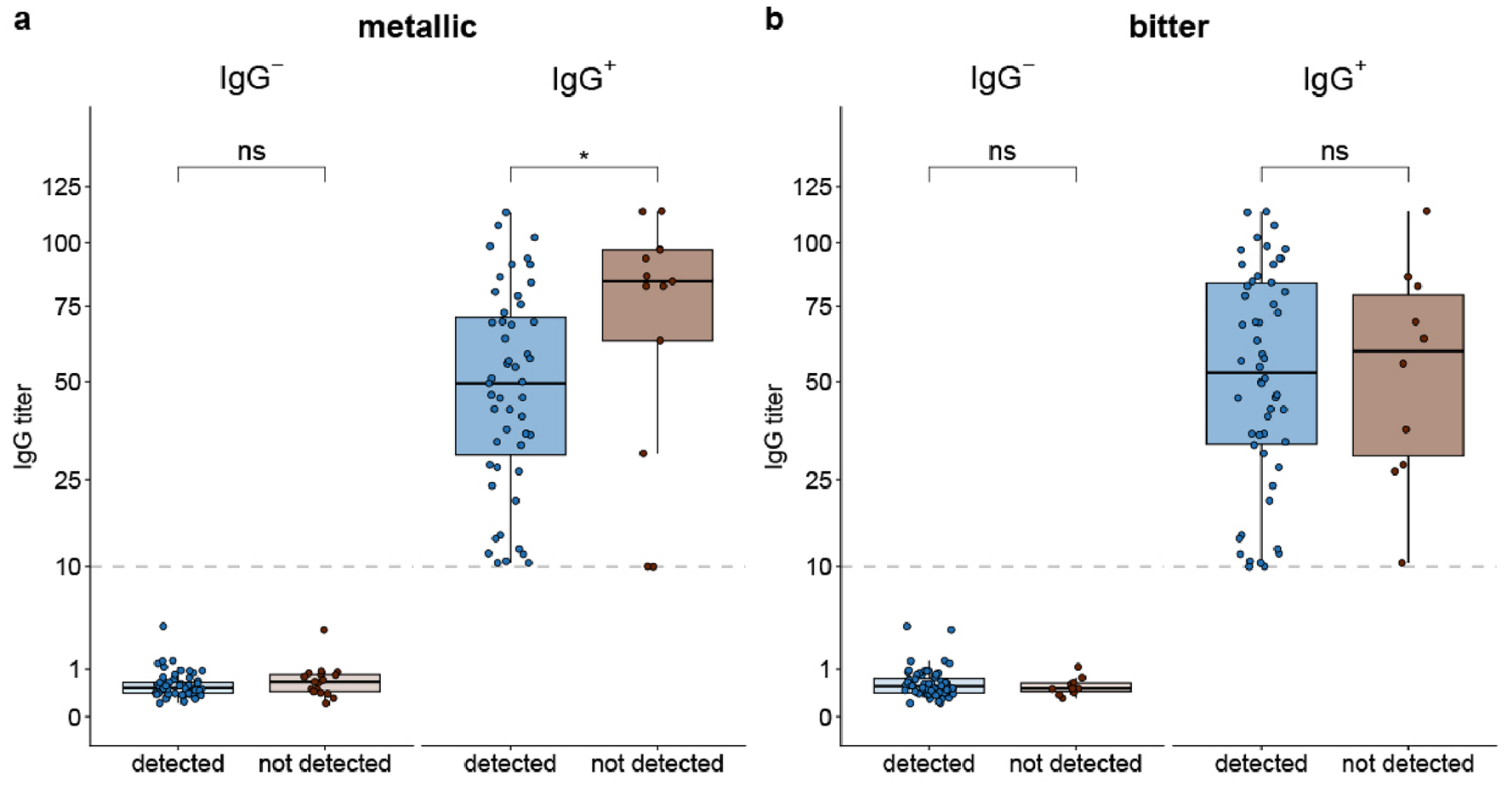
Ability to identify the taste qualities metallic and bitter depending on serum SARS-CoV-2 IgG levels. Participants performed a two-alternative forced choice task using test substance solutions and water for the taste qualities sweet, spiciness, sour, bitter, and metallic. Data are shown as box plot (median ± interquartile range). (**a**) IgG^+^ participants who did not detect metallic taste tested by iron(II)-gluconate (0.1 g/L) had higher IgG levels; (**b**) IgG levels were not different between participants with and without impaired bitter taste perception tested by caffeine (0.6 g/L).

The sweet solution was identified correctly by all IgG^−^ subjects and almost all of the IgG^+^ participants (98.4%; 63/64). The spicy solution was almost always identified correctly, as 97.4% (75/77) of IgG^−^ and 96.9% (62/64) of IgG^+^ participants could identify it. Sour instead of the water solution was chosen correctly by 95.3% (61/64) of IgG^+^ and 96.1% (74/77) of IgG^−^ patients. The metallic solution was detected by 79.7% (51/64) of IgG^+^ participants, whereas 77.9% (60/77) of the IgG^−^ participants identified the metallic solution correctly (Fig. 1a). Interestingly, IgG^+^ levels were higher in those participants who failed to detect metallic taste than in those who identified metallic taste correctly (*p* = 0.03) (Fig. 1a). The overall percentages of participants who did detect bitter correctly were 84.4% (54/64) in the IgG^+^ and 87.0% (67/77) in the IgG^−^ group (Fig. 1b), which did not correlate with the participant’s IgG titer.

### Transcriptome signatures of foliate papillae swabs

**Figure 2:**
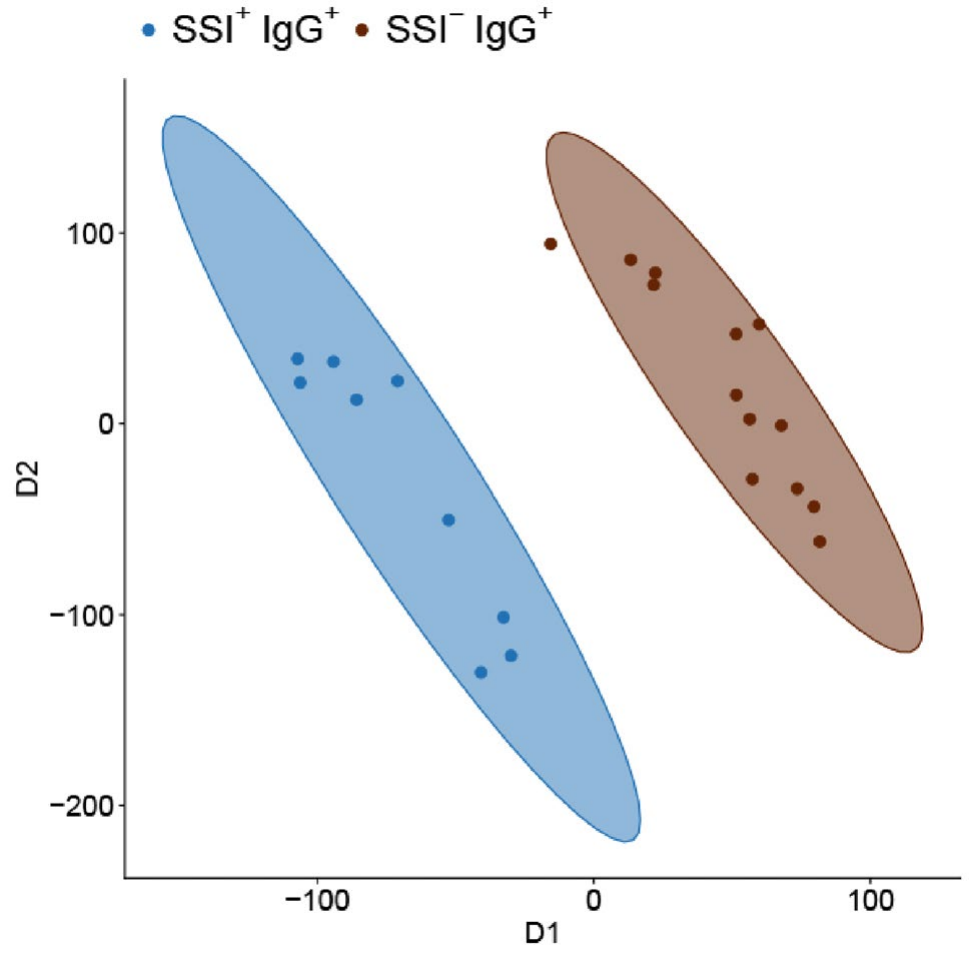
PLS-DA score plots of SARS-CoV-2 IgG^+^ participants separate the transcriptome signatures of SSI^+^ and SSI^−^.

To analyze whether the affected metallic taste perception in IgG^+^ participants can be attributed to alterations in sensory perception related genes, the transcriptome of tongue foliate papillae was investigated via microarray analyses. Participants were divided into four groups, depending on their self-reported sensory impairments at study start (SSI^±^) and SARS-CoV-2 IgG serostatus (IgG^±^) (Table 1). Raw data from scanned microarrays was normalized using the Robust Multichip Average (RMA) algorithm, while array data quality was evaluated using probe-level models. The resulting median deviations were visualized as relative log expression plot (see Supplementary Fig. S1), which confirms the low level of unwanted variation, as individual sample medians are located close to zero and similar interquartile ranges indicate an equal degree of dispersion. Since human cohorts differ in many variables, a supervised Partial Least-Squares Discriminant Analysis (PLS-DA) was performed to classify samples voluntarily donated by 43 participants of our cohort based on their transcriptome signatures from the tongue foliate papillae area. In the SSI^+^ group, the PLS-DA revealed different transcriptome signatures, depending on the SARS-CoV-2 status (see Supplementary Fig. S2). The results of the PLS-DA show a difference in the overall foliate papillae transcriptome signatures based on the variables of IgG status and SSI status. Notably, subjects SSI^+^ and SSI^−^ could be distinguished from each other within the two groups IgG^+^ vs. IgG^−^. The PLS-DA score plot highlights the clear separation between IgG^+^ participants with and without sensory disorders based on their whole genome transcription profile, which is also illustrated by the non-overlapping ellipses visualizing the 95% confidence intervals (Fig. 2). Similarly, in the subgroup of all IgG^−^ participants, the cases with and without smell and taste loss are separated (see Supplementary Fig. S3).

### RNA expression level of individual genes

**Figure 3:**
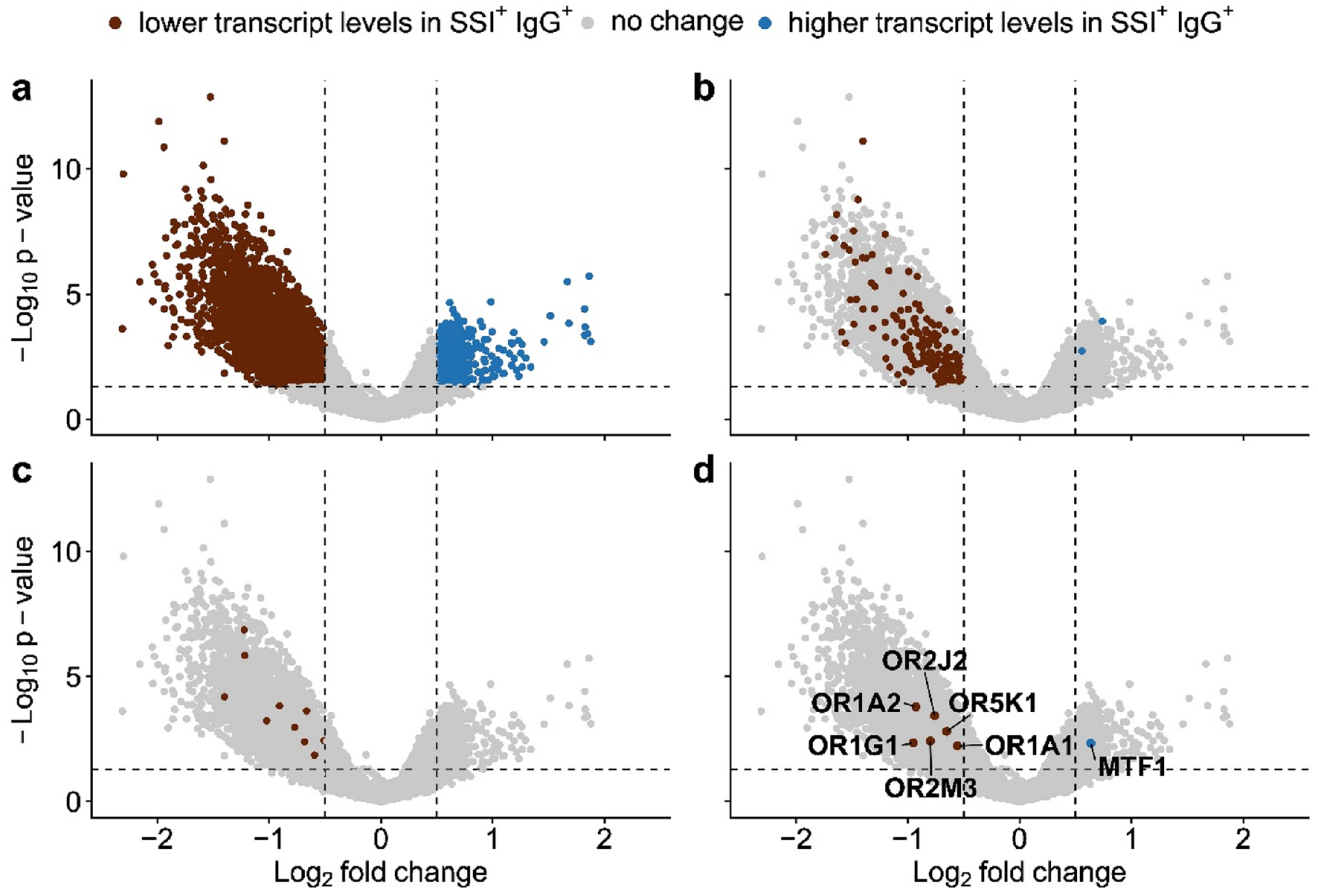
Volcano plot of the RNA transcript differences occurring specifically in SSI^+^ IgG^+^ participants. Each dot represents a specific gene. (**a**) 5356 genes with significantly (*p* < 0.05) lower transcript levels and a fold-change of ≥ 1.4 are highlighted in brown (left). 790 genes with significantly (*p* < 0.05) higher transcript levels and a fold-change of ≥ 1.4 are highlighted in blue (right). (**b**) Smell associated genes are highlighted. Overall, 166 olfaction associated genes had lower transcript levels (*p* < 0.05) and a fold-change of ≥ 1.4. (**c**) Taste associated genes are highlighted. Overall, 10 taste associated genes had lower transcript levels (*p* < 0.05) and a fold-change of ≥ 1.4. (**d**) Metal perception associated genes are highlighted. Six metal perception associated genes had lower transcript levels (*p* < 0.05) and a fold-change of ≥ 1.4. One metal perception associated gene had a higher transcript level (*p* < 0.05) and a fold-change of ≥ 1.4.

Smell and taste disorders are prevalent in different populations. A unique strength of this study set up with the four groups (SSI^±^ and IgG^±^) and the 43 microarrays used is that we adjusted^24^ the change between SSI^−^ IgG^+^ and SSI^+^ IgG^+^ samples for the general effect of a sensory disorder without a SARS-CoV-2 infection (SSI^+^ IgG^−^ vs. SSI^−^ IgG^−^). This enabled us to focus on changes in the RNA expression due to their SARS-CoV-2 infection that are apparent in the foliate papillae of SSI^+^ IgG^+^ participants. Overall, 790 genes displayed higher (*p* < 0.05) transcript levels and a positive fold-change higher than 1.4 in SSI^+^ IgG^+^ subjects (blue on the right in Fig. 3a). 5356 genes had lower (*p* < 0.05) transcript levels and a negative fold-change higher than 1.4 (brown on the left in Fig. 3a) compared to the other three groups of participants (IgG^+^SSI^−^, IgG^−^SSI^+^, IgG^−^SSI^−^).

### Gene ontology enrichment of the foliate papillae transcriptome

**Figure 4:**
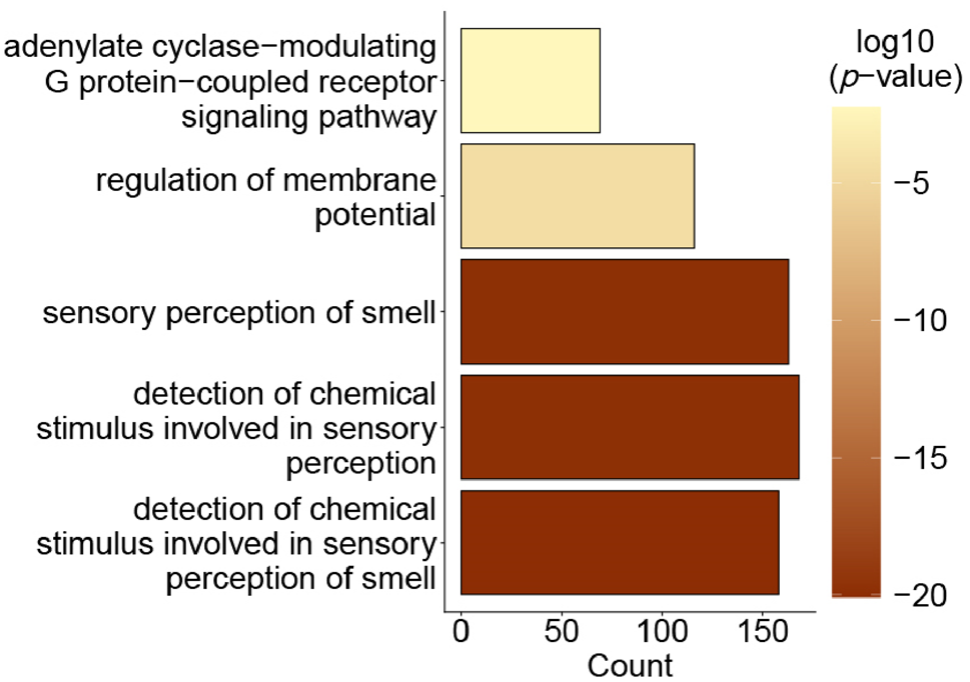
Biological process gene ontology over-representation analysis of specifically less transcribed genes in the foliate papillae area on the tongue of SSI^+^ IgG^+^ participants.

To investigate whether the 5356 genes with a lower RNA expression and a fold-change higher than 1.4 in SSI^+^ IgG^+^ subjects adjusted for the RNA transcript levels of the other three groups of participants (IgG^+^ SSI^−^, IgG^−^ SSI^+^, IgG^−^ SSI^−^) are associated with common biological processes, over-representation analysis based on gene ontology of the set of differentially expressed genes was performed^25^. This analysis yielded five significant results using the biological process ontology and a *p*-value of 0.05 as the cutoff following adjustment by the Benjamini–Hochberg procedure (Fig. 4, see Supplementary Fig. S4 and Supplementary Table S2). The gene ontology terms “detection of chemical stimulus involved in sensory perception of smell” (GO:0050911; 158/3120; adjusted *p*-value = 8.5E^−21^), “detection of chemical stimulus involved in sensory perception” (GO:0050907; gene ratio = 168/3120; adjusted *p*-value = 1.5E^−20^), “sensory perception of smell” (GO:0007608; gene ratio = 163/3120; adjusted *p*-value = 1.5E^−20^), “regulation of membrane potential” (GO:0042391; gene ratio = 116/3120; adjusted *p*-value = 3.7E^−5^), and “adenylate cyclase-modulating G protein-coupled receptor signaling pathway” (GO:0007188; gene ratio = 69/3120; adjusted *p*-value = 5.7E^−3^) were over-represented. This overrepresentation of genes involved in sensory perception corroborated our hypothesis. Thus, we further investigated smell and taste genes differentially expressed in the foliate papillae on the tongue of the participants.

### RNA expression level of genes associated with smell

Overall, 168 smell-associated genes displayed a changed transcript level and a fold-change higher than 1.4 in SSI^+^ IgG^+^ participants compared to the other three groups of participants (IgG^+^ SSI^−^, IgG^−^ SSI^+^, IgG^−^ SSI^−^) (Fig. 3b, see Supplementary Table S3). OR6C4 and olfactory marker protein (OMP) are the only two smell-associated genes which had higher transcript levels and a fold-change higher than 1.4 in SSI^+^ IgG^+^ subjects, whereas 166 olfactory receptor genes had significantly lower transcript levels and a fold-change higher than 1.4 in SSI^+^ IgG^+^ participants.

### RNA expression level of genes associated with taste

In an assessment of genes associated with a function in sensory perception of taste (Fig. 3c) defined by gene ontology enrichment, 10 genes (see Supplementary Table S4) showed lower transcript levels and a fold-change higher than 1.4 in the foliate papillae of the SSI^+^ IgG^+^ subjects. Eight of these genes encode for receptors that belong to the bitter-sensing TAS2R family. Moreover, TAS1R1 had lower transcript levels and a fold-change higher than 1.4, which is a receptor involved in umami taste, and dimerizes with TAS1R3 to reach its full function.

### RNA expression level of genes hypothesized to be associated with sensory metal perception

Since the mechanisms of metal perception are still under scientific debate^20^, we investigated whether the observed impairment of metal perception in IgG^+^ participants in our sensory experiment correlates with decreased RNA transcript levels of metal perception-related genes. Genes associated with metal perception were collected from databases and the literature (see Supplementary Table S5). The metal regulatory transcription factor 1 (MTF1) showed higher transcript levels in the SSI^+^ IgG^+^ participants compared to the three control groups (Fig. 3d, see Supplementary Table S6). Lower transcript levels are apparent for OR2M3. The activation of this narrowly tuned thiol receptor is potentiated by copper binding^26^ (Fig. 3d, see Supplementary Table S6). Five olfactory receptors which can be associated with metal perception due to their ligand profile have lower transcript levels on the foliate papillae of these subjects (Fig. 3d, see Supplementary Table S6). One of the specialized olfactory receptors, OR5K1^27^, known to be targeted by metal-complexing pyrazines^20,28^, demonstrated lower transcript levels in SSI^+^ IgG^+^ participants. Due to their broad, metallic perceived agonist profiles, the four olfactory receptors OR1A2, OR2J2, OR1A1 and OR1G1 can be associated with metallic perception, and also showed lower mRNA expression levels in our study.

## Discussion

The aim of this study was to associate sensory impairments due to a preceding SARS-CoV-2 infection, evidenced by IgG levels ≥ 10 AU/mL (IgG^+^), with changes of the foliate papillae transcriptome in subjects with self-reported sensory impairments (SSI^+^) compared to study participants showing IgG levels ≤ 10 AU/mL (IgG^−^) and no self-reported sensory impairments (SSI^−^). Considering that smell and taste disorders are prevalent in most populations and most individuals recover their sense of smell and taste within a few days, a unique strength of this study setup are the four groups of participants (± self-reported sensory impairment (SSI^±^) and ± SARS-CoV-2 IgG (IgG^±^)). This allowed us to adjust for the non-COVID-19 and non-smell/taste disorder-related changes in the RNA expression. Finally, the investigated smell and taste disorders in our cohort enabled us to link transcriptome changes of participants with their smell/taste disorders due to the SARS-CoV-2 infection.

Cazzolla et al. 2023 report a relationship between dysfunctional taste of the grouped taste disorders of umami, bitter, and sweet (GPCRs) and IL-6 blood levels in moderate vs. mild current SARS-CoV-2-positive cases with taste disorders, while the sour and salty disorder group was not dependent on the IL-6 levels of the patients^29^. Although this describes the correlation of SARS-CoV-2 disease severity with taste disorders, no individual taste qualities were analyzed. Salty taste was not studied in our cohort because we focused more on likely unpleasant taste sensations such as bitter, sour, spicy, and metallic and included sweet as a pleasant comparator. Moreover, especially salty taste is impacted by a multitude of confounding factors, like the composition and flow of saliva^30,31^ and blood pressure^32^. In the forced choice sensory taste tests of our study, most participants correctly identified sweet, sour, and spicy taste qualities. For the detection of bitter taste, there was no difference in the percent IgG^+^ and IgG^−^ participants reporting the identification of bitterness, indicating that the bitterness from caffeine is independent of the IgG status. IgG levels were higher in IgG^+^ participants with impaired perception of metallic tasting iron gluconate than in IgG^+^ participants who detected metallic taste correctly. Since iron promotes a strong retro-nasal metal perception^18,21^, this result suggests olfactory involvement in the revealed metal taste distortion. However, it has to be noted that the self-report of chemosensory dysfunctions is hypothesized to under-represent the prevalence of impairments^16,33,34^ and is prone to subjective bias ^35^. It also has to be kept in mind that loss of flavor sensations from decreased retro-nasal olfactory stimulation is often reported as loss of taste ^36,37^. Concerning different food groups, no difference in sensory perception alterations for SSI^+^ IgG^±^ participants was identified. Thus, the same food groups seem to be equally affected by the changed perception, independent of the cause of chemosensory disorder. A unique strength of our study design is the use of whole transcriptome microarrays for the same cohort as the sensory cohort of this study. This enabled us to specifically look into the changes in the RNA transcriptome that are distinctive for the foliate papillae of SSI^+^ IgG^+^ participants compared to the three control groups. In this study, we selected the foliate papillae-rich area of the tongue for two reasons. First, this area is quite defined, which ensures reasonable reproducibility if tongue swabs have to be taken from different lab personnel. Second, we kept in mind recent reports on the impact of the oral microbiota on taste perception ^38^ and selected the foliate papillae-rich area of the tongue where bacterial coating is minimized ^39^. Moreover, the potential bias of bacterial DNA contamination was minimized by this approach ^40^. Overall, 5356 genes had significantly lower RNA expression levels and a fold change greater than 1.4, while only 790 genes had significantly higher RNA transcript levels and a fold change greater than 1.4 in SSI^+^ IgG^+^ subjects. Thus, the SARS-CoV-2 infection results in more genes being down- than upregulated in our study. The three most significant gene ontology enrichment terms (detection of chemical stimulus involved in sensory perception of smell, detection of chemical stimulus involved in sensory perception, and sensory perception of smell) strongly point to the biological function of smell. Furthermore, both taste and smell receptors belong to the superfamily of adenylate cyclase-modulating G protein-coupled receptor signaling pathway, which was also identified as being downregulated after SARS-CoV-2 infection by the gene ontology enrichment analysis. Olfactory receptors are not only expressed in the olfactory epithelium, but also peripherally ^41^. Furthermore, their function can be versatile. For example, in a liver cancer cell line, OR1A2 signaling was reported to influence proliferation ^42^. Co-expression of olfactory receptors with ACE-2 and TMPRSS2 is reported for the lung, esophagus, salivary gland, colon, testis, thyroid, kidney, heart, pancreas, and adipose tissue ^43^. Thus, the observed lower transcript levels of olfactory receptors might have heterogeneous consequences in different tissues and be relevant to the systemic response after a SARS-CoV-2 infection, including possible dysregulation of multiple physiological processes persisting after the acute phase of the infection. The result of lower transcript levels of olfactory receptors due to a SARS-CoV-2 infection is in accordance with the findings by Zazhytska et al. (2022), who performed RNA-seq analysis on olfactory epithelia obtained from 19 SARS-CoV-2 positive and 3 control subjects at the time of death, as well as time-dependent experiments on early stages of infection in golden hamsters. They described a widespread downregulation of olfactory receptors and their signaling cascade components in the nose in the acute phase ^44^), while we found lower olfactory receptor transcript levels in the foliate papillae on the tongue of participants long after the acute infection. Our observation of decreased mRNA expression levels of olfactory receptors on the foliate papillae, potentially as a sequela of SARS-CoV-2 infection, further raises the question of the functionality of these receptors in the foliate papillae. Malik et al. hypothesized that smell and taste might initially be integrated into the taste cells. In their experiments, olfactory receptors and their transduction molecules were expressed and responded to odorants in the taste papillae of green fluorescent protein-expressing transgenic mice and human fungiform taste papilla (HBO) cells, which could be inhibited by knockdown of adenylyl cyclase mRNA by specific small inhibitory RNA and pharmacological block of adenylyl cyclase ^45^. Moreover, ACE-2 has been reported to be expressed in human type II taste cells ^46^ and HBO cells ^47^. Thus, the lower transcript levels of the olfactory receptors demonstrated here can be hypothesized to have a functional consequence on the tongue. This suggests that the odorant receptors on the tongue might play a functional role in chemosensory perception and that they are potential targets for virus-initiated loss of taste and smell. TAS1R1 is a taste receptor involved in umami taste, which dimerizes with TAS1R3 for its full function in detecting several amino acids, such as ʟ-glutamate and 5′-ribonucleotides ^48,49^. Its observed downregulation in this study hints at a potentially reduced umami taste perception. The decreased expression levels of 8 TAS2R bitter receptors, especially the lower levels of the TAS2R7 RNA transcript, which is reported to play a role in metal perception ^50^, align with our sensory taste experiments. These two results point towards a potential association between reduced bitter and metallic sensation after a SARS-CoV-2 infection due to the downregulation of bitter taste receptors. The perception of smell or taste is often multimodal and hard to differentiate precisely. Metal-responsive transcription factor-1 (MTF1) had higher RNA expression levels in SSI^+^ IgG^+^ participants. The pluripotent transcriptional regulator MTF1 is important for metal homeostasis and stress responses, primarily exposure to heavy metals but also hypoxia or oxidative stress ^51^. Thus, a higher MTF1 mRNA expression level due to a SARS-CoV-2 infection is a possible stress response. Copper has been reported to potentiate the receptor activation of OR2M3 when the specific ligand 3-mercapto-2-methylpentan-1-ol is present and concentration-dependently inhibited a constitutive activity of the receptor without the ligand ^26^. The decrease of OR2M3 transcript levels in our study in SSI^+^ IgG^+^ subjects might be the starting point for further research in the roles of metal sensitive olfactory receptors. OR5K1 had decreased transcript levels in this study and is known to detect pyrazines ^27^. Pyrazines contribute to the roasted, metallic or earthy aroma of foods and are formed during the heating of food as Maillard products^52–54^. Furthermore, metals occur naturally or are added to food, i.e. to enhance their flavor during heating, building complexes with pyrazines^55,56^. The role of pyrazines in metallic perception needs to be determined, as the decreased expression levels of this specialized receptor might contribute to the observed decrease of metallic perception. Due to their agonist profiles, we hypothesize that the four olfactory receptors OR1A2, OR2J2, OR1A1 and OR1G1 are associated with metallic perception, which had lower mRNA expression levels and a fold-change greater than 1.4 in our study. The broadly tuned OR1A1 preferably responds to esters, acetates, ketones, terpenes, sulfur-containing compounds, aldehydes, alcohols, and lactones^57^. The strongest agonists reported were (R)-(−)-carvone, (R)-(+)-limonene, 2-phenylethanethiol, (S)-(−)-limonene and 3-mercaptohexyl acetate^57^. For OR1A2, citronellic terpenoid and aliphatic aldehydes of a carbon chain length C7–C8 were suited best as ligands^58,59^. Coumarin, 1-octanol, 1-heptanol, 1-nonanol, 1 decanol are reported as ligands for OR2J2^42,59^. For OR1G1, the most active odorants were alcoholic, ester, lactone and aldehyde, ketones of a carbon chain length of C8–C10^60^. Thus, these receptors are broadly tuned and can detect alcohols, aldehydes and ketones with chain lengths from 7 to 10 carbon atoms. These ligands of our hypothesized receptors are reported in the context of metallic sensations. Odorous aldehydes are formed due to lipid oxidation after iron solution ingestion in the oral cavity and are one of the mechanisms leading to metallic sensation^61^. C6 to C10 *n*-alkanals, unsaturated aldehydes and ketones are formed in this process, among which 1-octen-3-one was discussed to be a key odorant, for metallic smell in skin in contact with iron^62^ and oxidized apple juice^21,62,63^. Further, alk-1-en-3-ols and alk-1-en-3-ones of chain length six- and seven-carbons are reported to smell metallic^64^. Metallic smelling oxidation products are off-flavors in several food items that have these metallic smelling volatiles in their profile, such as mushrooms, soybean oil, olive oil, artichoke, honey, dill, butter oil, and grapefruit juice^64^. In olive oil, metallic smell was reported for the volatile trans-4,5-epoxy-(E)-2-decenal^65^. Thus, the decreased expression levels of OR1A2, OR2J2, OR1A1, and OR1G1 might contribute to the observed impairment of the identification of the metallic taste solution. As these receptors are broadly tuned, their down-regulation might have a wide impact, and future studies are needed to confirm this hypothesis. The functional role of odorant receptors in sensory metal perception needs to be verified, e.g. by knock-out/down experiments^66–68^. Finally, not many receptors and ligands are known to contribute to metallic perception. Thus, the insights gained from this study may be of assistance to unravel ligand-based mechanisms of metal perception.

As this study was conducted with Munich hospital personnel, selection bias might limit the results of this study. In this study, all courses of disease were mild, without the need for hospitalization. Thus, our population did not generally represent the course of the disease and the average demographic population. Despite the mild courses, the intensity and duration of symptoms were notable. The 158 participants were matched for age, sex, and occupational SARS-CoV-2 exposure risk. Inadequately balanced sample sizes might have impacted our outcomes. The self-reported sensory impairments might be prone to recall bias. Further, our samples were collected August 2020 to September 2020 in the first wave of the pandemic in Germany. Newer SARS-CoV-2 variants may not be resulting in identical chemosensory alterations.

In conclusion, this study set out to find sensory impairments due to a former SARS-CoV-2 infection that could be verified with standardized sensory tests, as well as to establish links between these sensory impairments and gene expression changes in the foliate papillae area of the tongue. A high SARS-CoV-2 IgG titer was accompanied by disorders in metallic iron gluconate perception post infection. Further research might explore if metal perception is also impaired due to infections with other viruses also known to cause olfactory dysfunctions. Whether sensory impairments identified by tests based on specific food items with metallic tasting/smelling properties could be indicative of the course of a SARS-CoV-2 infection needs to be verified in future studies. The results of this investigation identified decreased mRNA expression levels of 166 olfactory receptors on the foliate papillae area of the tongue in IgG^+^ SSI^+^ participants. On the one hand, this leads to the question of whether these receptors might play a role in perception on the tongue through an interplay with taste receptor pathways. On the other hand, much research is needed to investigate other possible functional roles beyond perception for these widely distributed receptors. Especially for investigating the blind spot of metal perception, the lower transcript levels of OR1A2, OR2J2, OR1A1, OR5K1 and OR1G1, as well as TAS2R7 discussed in this study, may be of assistance to unravel ligand-based mechanisms.

## Methods

### Sample collection

Between April 14, 2020 and May 29, 2020, after the first wave of the pandemic, 4554 employees of the university hospital *Klinikum rechts der Isar* and medical students at the Technical University of Munich (TUM) were tested for SARS-CoV-2 immunoglobulin G (IgG) in the prospective, monocentric, observational SeCoMRI-study^22^. Our participants were classified as SARS-CoV-2 IgG positive or negative as in the preceding SeCoMRI-study^22^. Accordingly, seropositivity of our patients was classified by a combination of two out of the following three diagnostic tests: (i) IgG against SARS-CoV-2 spike 1 protein or nucleocapsid protein by using a paramagnetic particle chemiluminescent immunoassay on an iFlash 1800 Immunoassay Analyzer (Shenzhen Yhlo Biotech Co, Shenzhen, China), with IgG (> 10 AU/mL) considered as seropositive (ii) total antibodies against SARS-CoV-2 nucleocapsid by using an electro-chemiluminescent immunoassay on a Cobas e411 Analyzer (Roche Diagnostics, Basel, Switzerland), or (iii) IgG against SARS-CoV-2 spike 1 protein by using an ELISA (Euroimmun, Luebeck, Germany)^22^.

This study is in accordance with the Declaration of Helsinki and was approved by the Ethics Committee of the Technical University of Munich, School of Medicine, approval number: 216/20 S. All study participants provided informed consent for this study which allowed re-contact for further studies. 158 participants were successfully recruited for the study for smell and taste loss. Baseline sample collection was performed from August 2020 to September 2020, which was a median 3 months after SARS-CoV-2 IgG detection. According to the SARS-CoV-2 IgG serostatus (IgG^±^) and the presence or absence of self-reported sensory impairment (SSI^±^), 4 groups of participants were assigned. All 158 participants underwent a questionnaire (see Supplementary Questionnaire S1). 141 subjects consented to sensory testing (see Supplementary Questionnaire S2). Additionally, 43 RNA samples from swabs of the foliate papillae region were collected. The participation and grouping of subjects in the different study sections are displayed in Table 2. A questionnaire was used at the visit to determine subjective alterations in smell and taste, focusing on prevalence and duration, intensity, and influence on food consumption, especially for coffee, tea, chocolate, cheese, meat products, fruits, vegetables, and bread. Subjects were requested to describe their smell/taste disturbance on a numeric rating scale with *x* = 0 meaning no disturbance and *x* = 10 meaning highest possible disturbance (see Supplementary Questionnaire S1). To quantify alterations in their sense of taste, 141 participants were subjected to sensory testing on the qualities sweet, sour, bitter, metallic, and spiciness, using solutions containing the substances listed in Table 3 (see Supplementary Questionnaire S2). All sensory test substances fulfill the criteria for food additives, as regulated in EU Nr. 231/201211, Nr. 1334/200812, in the German regulation on authorization of food additives (Zusatzstoff-Zulassungsverordnung, ZZulV), and in the regulation on specification and purity criteria (Zusatzstoff-Verkehrsverordnung, ZVerkV). The solutions were prepared using mineral water with a low mineral content. To prevent the effects of oxidation, fresh solutions were prepared daily. Participants were offered, in a two-alternative forced choice setup (see Supplementary Questionnaire S2) for each individual taste quality, two 20 mL samples (A and B) in plastic cups, one containing the tasting solution and the other containing plain water, as used prior for preparation. Following a single-blind procedure, participants were informed about the quality tested but not about the substance used in the solutions. Participants were instructed to follow a sip-and-spit protocol and to indicate subsequently the sample having a more intense taste.

**Table 3:** Test substances used in the sensory tests.

Quality	Substance	Chemical Abstract Service (CAS) Nr	Concentration in water at room temperature (g/L)
Sweet	Sucrose	57-50-1	10
Sour	Citric acid	77-92-9	0.6
Bitter	Caffeine	58-08-2	0.6
Metallic	Iron(II)-gluconate-hydrate	699014-53-4	0.1
Spiciness	Nonivamide	2444-46-4	0.001

### RNA extraction of tongue swab material

On the day of their study visit, tongue swabs of the foliate papillae area on the posterior edge of the tongue, where many taste buds are located, were obtained. The swabs (Copan, Brescia, Italy) were rubbed on the foliate papillae-rich area of the participant's tongue with a moderate intensity and then stored in Aimes medium at − 80 °C according to the manufacturer's instructions until further processing. RNA was prepared following the instructions of the Monarch Total RNA Miniprep Kit (New England Biolabs, Frankfurt am Main, Germany) for swabs. RNA concentration was measured via NanoDrop One (Thermo Scientific, Schwerte, Germany). Using the Concentrator Plus (Eppendorf, Hamburg, Germany), the volume of the RNA extract was reduced to approximately 5 µL.

### Reverse transcription with labeling

RNA was reverse transcribed and labeled by using SuperScript IV First-Strand Synthesis System (Invitrogen by Thermo Fisher Scientific, Vilnius, Lithuania), 5-propargylamino-dCTP-Cy3 (Jena Bioscience, Jena, Germany) and Cy3-labeled random nonamer primers (Tebu Bio, Offenbach, Germany) as described by Ouellet et al.^69^. After the samples were neutralized with 60 µL 1 M HEPES pH 7, they were purified with QIAquick purification kit (Qiagen, Hilden, Germany). The ssDNA and Cy3 concentrations were measured using NanoDrop One. The samples were dried completely with the Concentrator Plus (Eppendorf, Hamburg, Germany) and frozen at − 80 °C.

### Transcriptome analysis

Whole genome cDNA microarrays, which additionally included multiple 60mer oligonucleotide probes of genes predominantly associated with G-protein-coupled receptors, were synthesized and used to quantify gene expression as described previously^67,70,71^. A volume of 0.13 μL herring sperm DNA (10 mg/mL), 0.7 μL acetylated BSA (10 mg/mL), 6 μL 2 × MES buffer, 0.45 μL QC25 (Cy3-labeled, 100 nM), 0.45 μL ECO1BioA1 (Cy3-labeled, 100 nM), 0.45 μL ECO1BioD2 (Cy3labeled, 100 nM), and Cy3-labeled cDNA in ~ 4 μL RNase-free water (ca. ~ 40 ng) were combined for the hybridization mix. The solution (12 μL total) was pipetted onto an 18 mm × 18 mm coverslip (Nexterion Glass D Coverslip, 1098576) and the microarray, synthesis area facing down, lowered onto the coverslip until surface tension brought the two surfaces into contact. A hybridization chamber (Grace Bio-labs) was placed around the coverslip with the pipetting ports left open and the microarray, synthesis area facing up, was hybridized at 42 °C for 24 h while floating on a water-filled Petri dish without rotation. In the pre-warmed non-stringent wash buffer (SSPE; 0.9 M NaCl, 0.06 M phosphate, 6 mM EDTA, and 0.01% Tween20), microarrays were then washed for 2 min, in the stringent wash buffer (100 mM MES, 0.1 M Na^+^, and 0.01% Tween20) for 1 min, and for about 5 s in the final wash buffer (0.1 × saline-sodium citrate buffer). Drying was performed in a microarray centrifuge. The microarrays were subsequently scanned with an Axon GenePix 4400A microarray scanner (Molecular Devices, Sunnyvale, CA, USA) at 2.5 μm resolution. Fluorescence intensities were analyzed using NimbleScan 2.1 software (Roche NimbleGen).

### Statistics

Data analysis was performed using the statistical programming environment R (version 4.1.0^72^). Statistical differences in the IgG titer between panelists being able or not to detect the individual taste qualities were evaluated using robust testing strategies implemented in the R extension package WRS2^73^. As Levene’s test showed significant *p*-values, suggesting that there are significant differences between the variances of the perception groups, the two-sample trimmed mean test proposed by Yuen^74^, which allows for the presence of unequal variances, was conducted.

For analysis of data from cDNA microarrays, a custom annotation and design package was developed consisting of a SQLite database containing feature-level data such as x, y position on the array as well as feature set IDs. Subsequently, xys-files obtained from NimbleScan 2.1 software (Roche NimbleGen) could be directly imported into R followed by background correction and normalization using robust multichip average (RMA)^75^ and probe level models (PLM). Quality of the arrays and the preprocessing was accessed by inspection of the normalized data of the control probes added in the hybridization mix (QC25, ECO1BioA1, ECO1BioD2). Mapping of individual probe IDs to the Ensemble database was performed by application of the R extension package biomaRt^76^. For multivariate sample classification, sparse Partial Least Squares—Discriminant Analysis (sPLS-DA) models^77^ were trained using fivefold cross validation and 100 repeats. Visualization of binary group differences was performed by volcano plots after calculation of fold changes (> 1.4) and significance levels using the R extension package limma^78^ and a 2 × 2 factorial design with treatment-contrast parametrization, while ggplot2^79^ was used for plot generation. Gene ontology over-representation analysis was conducted using the enrichGO function implemented in the clusterProfiler package^80,81^ and the following parameters: OrgDb = org.s.eg.db, ont = “BP”, pAdjustMethod = Benjamini-Hochberg, pvalueCutoff = 0.01, qvalueCutoff = 0.01. Metallic genes included in the analysis are displayed in Supplementary Table S5.

### Supplementary Information

Supplementary Information.

## Data Availability

The data generated during this study are not publicly available due to privacy reasons, but are available from the corresponding author (please contact Veronika Somoza, v.somoza.leibniz-lsb@tum.de) on reasonable request.
