# Supplementary material for: Impaired metal perception and regulation of associated human foliate papillae tongue transcriptome in long-COVID-19
Source: Sci Rep. 2024 Jul 4;14:15408. doi: 10.1038/s41598-024-66079-w (PMC11224223; doi:10.1038/s41598-024-66079-w)
Supplement: Supplementary file 1 — Supplementary Information. [file 41598_2024_66079_MOESM1_ESM.pdf]

Supplementary material

Supplementary questionnaires

Supplementary translated questionnaire S1 (Visit 1/1)

Do you have alterations in your sense of smell or sense of taste? Depending on this answer please fill the appropriate column of the table.

☐ Yes	☐ No
1. For how long have your alterations in sense of smell persisted?<br><br>• 1 week ☐<br>• 2 weeks ☐<br>• 4 weeks ☐<br>• more than 4 weeks ☐	Did you have alterations in sense of smell or sense of taste in the time between your SeCoMRI-Visit and today?<br><br>☐ No<br><br>☐ Yes:<br><br>☐ Before the SeCoMRI-visit:

2. For how long have your alterations in sense of taste persisted?

- 1 week ☐
- 2 weeks ☐
- 4 weeks ☐
- more than 4 weeks ☐

3. How intense are your alterations in sense of smell on a scale from 0 (none) to 10 (high intensity)?

4. How intense are your alterations in sense of taste on a scale from 0 (none) to 10 (high intensity)?

1. For how long did your alterations in sense of smell persist?

- 1 week ☐
- 2 weeks ☐
- 4 weeks ☐
- longer than 4 weeks ☐

2. For how long did your alterations in sense of taste persist?

- 1 week ☐
- 2 weeks ☐
- 4 weeks ☐
- longer than 4 weeks ☐

3. How intense were your alterations in sense of smell on a scale from 0 (none) to 10 (high intensity)?

4. How intense were your alterations in sense of taste on a scale from 0 (none) to 10 (high intensity)?

5. Consuming which food or nutriment did you notice most your alterations in **sense of smell** and/or **smell of taste?**

6. Please evaluate the intensity of your alterations in **sense of smell** and/or **sense of taste** when consuming the following foods:

Food	Strong alterations	Weak alterations	No alterations	I never/rarely consume
Coffee				
Tea				
Chocolate				
Cheese				
Meat products				
Vegetables				
Fruit				
Bread				

Supplementary translated questionnaire S2 (V1/2)

Which of the two solutions has a more intense taste, corresponding to the given quality? Should you perceive both solutions as equal, please select a random answer!

<u>Test-Pair 1: sweet</u>

- 1A ☐
- 1B ☐

<u>Test-Pair 2: sour</u>

- 2A ☐
- 2B ☐

<u>Test-Pair 3: bitter</u>

- 3A ☐
- 3B ☐

<u>Test-Pair 4: metallic</u>

- 4A ☐
- 4B ☐

<u>Test-Pair 5: pungent</u>

- 5A ☐
- 5B ☐

Supplementary Figures

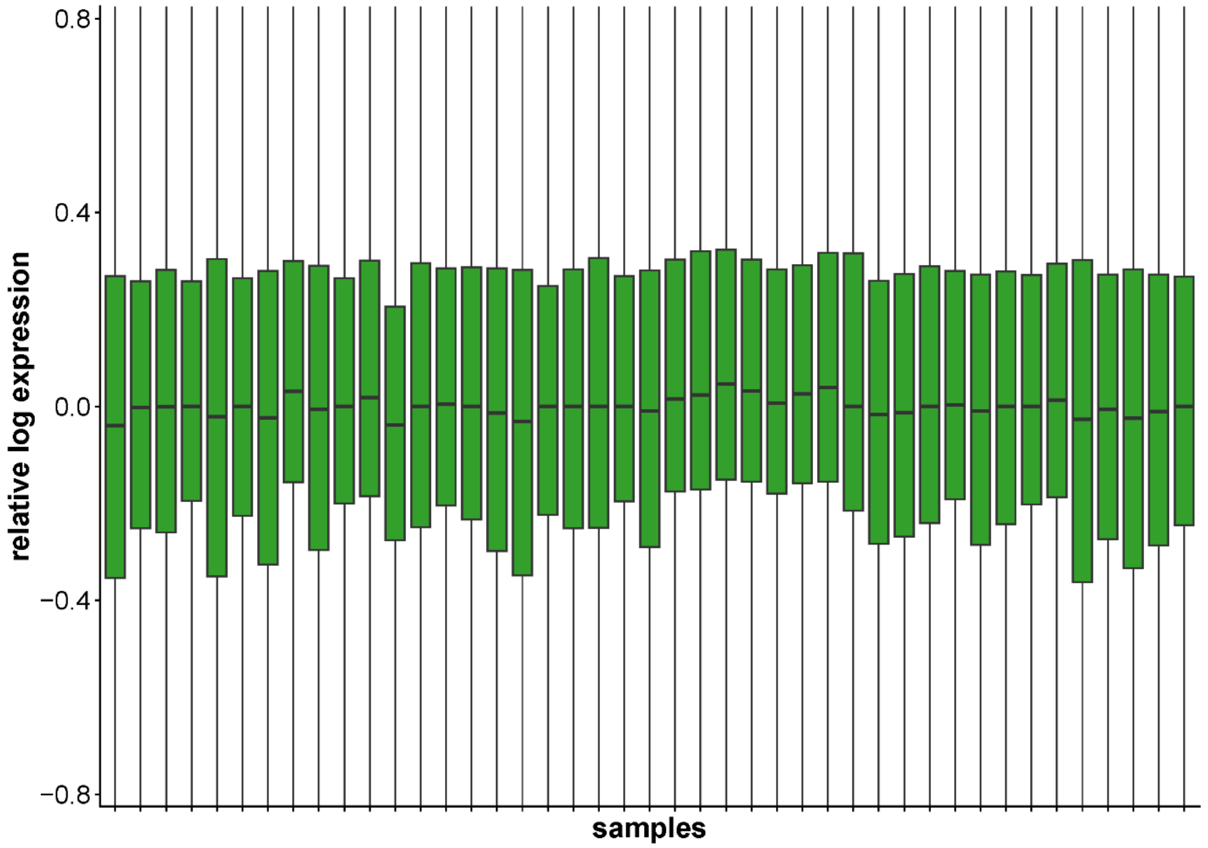

Supplementary Fig. 1 Relative log expression plot highlighting the comparable data quality of individual microarrays.

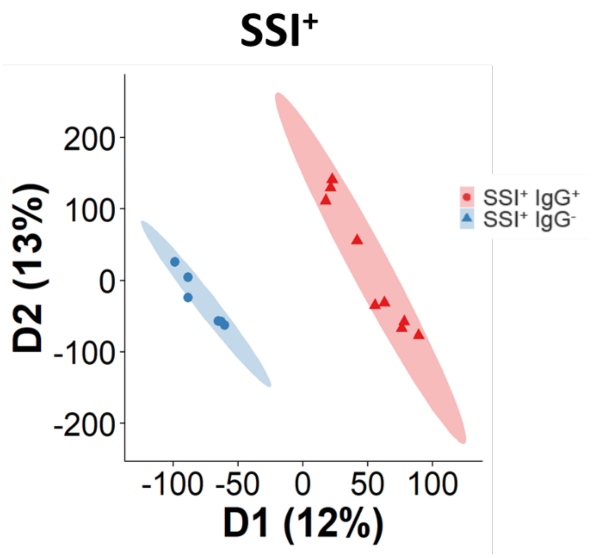

Supplementary Fig. S2 PLS-DA score plot for SSI^{+} participants. The PLS-DA revealed different transcriptome signatures depending on IgG$^{\pm}$ status.

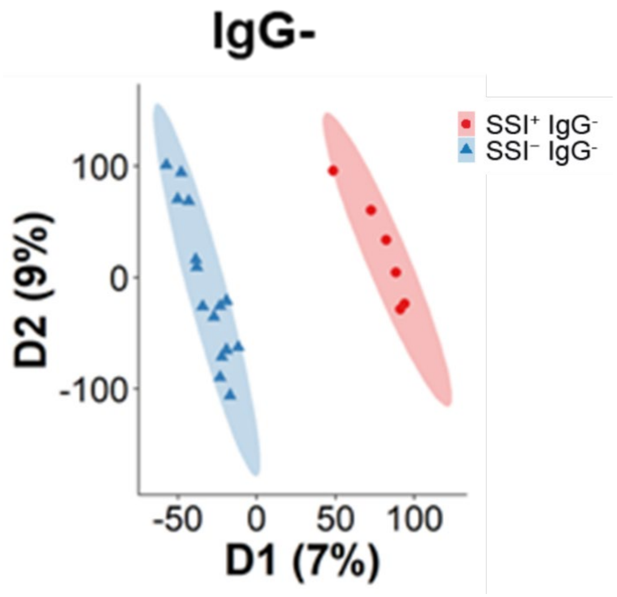

Supplementary Fig. S3 PLS-DA score plots of IgG^{-} participants separate the transcriptome signatures of the SSI$^{\pm}$ cases.

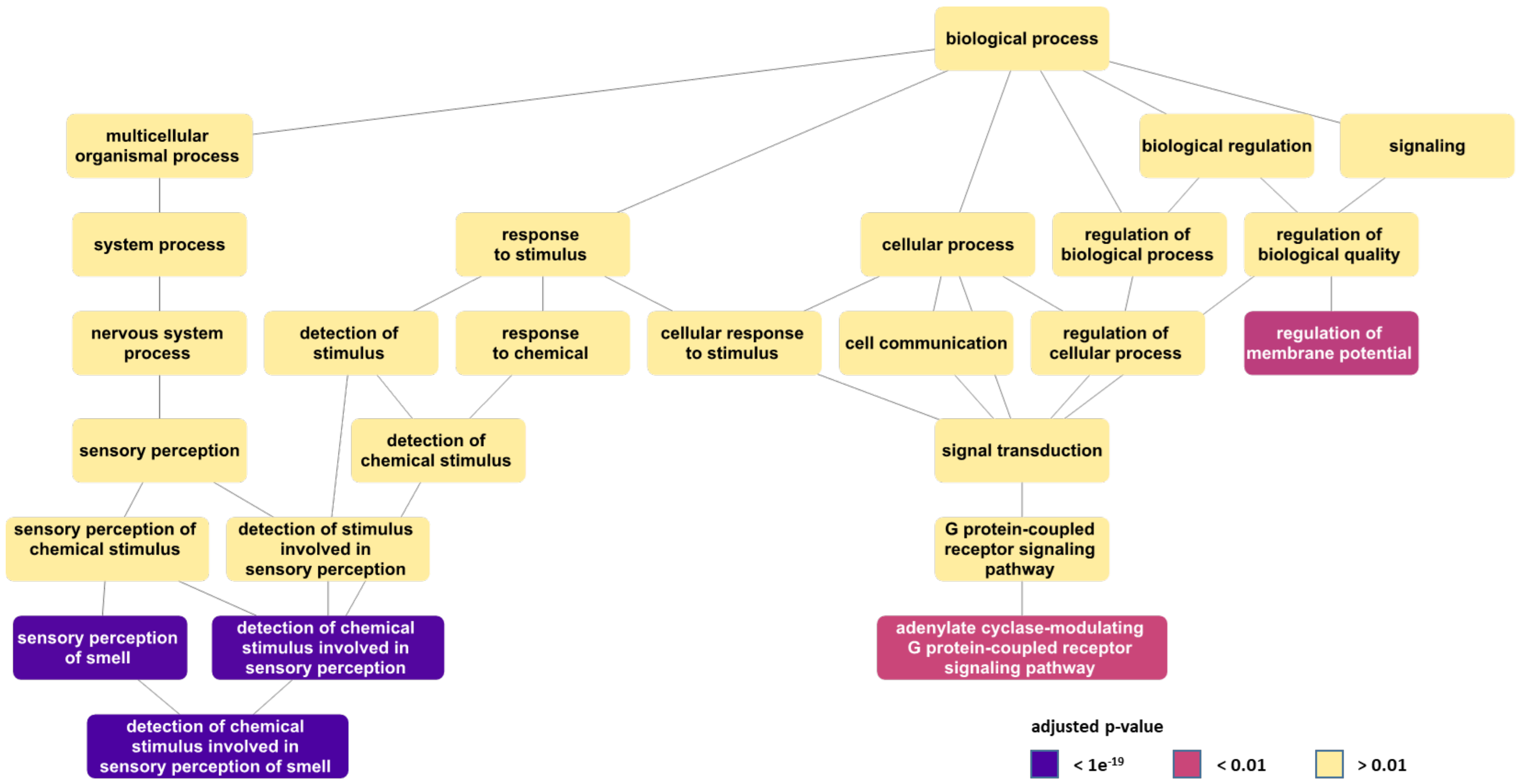

Supplementary Fig. S4 **Biological process gene ontology over-representation analysis of specifically less transcribed genes in the foliate papillae area on the tongue of SSI^{+} IgG^{+} participants.**

Supplementary Tables

Supplementary Table S1 Self-reported sensory alterations of food groups in SSI$^+$ participants per IgG$^\pm$ (38 participants per IgG group):

	SARS-CoV-2 seronegative with smell/taste disturbances *n* (%)			SARS-CoV-2 seropositive with smell/taste disturbances *n* (%)		
	**sensory alterations**	**no sensory alterations**	**not consumed**	**sensory alterations**	**no sensory alterations**	**not consumed**
**Coffee**	25 (66%)	9 (24%)	4 (11%)	21 (55%)	12 (32%)	5 (13%)
**Tea**	17 (45%)	10 (26%)	11 (29%)	13 (34%)	14 (37%)	11 (29%)
**Chocolate**	22 (58%)	9 (24%)	7 (18%)	21 (55%)	11 (29%)	6 (16%)
**Cheese**	26 (68%)	10 (26%)	2 (5%)	20 (53%)	13 (29%)	5 (13%)
**Meats**	22 (58%)	6 (16%)	10 (26%)	19 (50%)	14 (34%)	5 (13%)
**Vegetables**	27 (71%)	9 (24%)	2 (5%)	24 (63%)	12 (32%)	2 (5%)
**Fruits**	25 (66%)	13 (34%)	0 (0%)	19 (50%)	17 (45%)	2 (5%)
**Bread**	23 (61%)	12 (32%)	3 (8%)	16 (50%)	19 (50%)	3 (8%)

Supplementary Table S2 Biological process gene ontology over-representation analysis of less transcribed genes on the tongue of SSI$^+$ IgG$^+$ participants.

ID	Description	Gene Ratio	BgRatio	*p*-value	Adjusted[19] *p*-value	*q*-value
GO:0050911	detection of chemical stimulus involved in sensory perception of smell	158/ 3120	431/ 18903	1.4E-24	8.5E-21	8.2E-21
GO:0007608	sensory perception of smell	163/ 3120	457/ 18903	7.3E-24	1.5E-20	1.5E-20
GO:0050907	detection of chemical stimulus involved in sensory perception	168/ 3120	477/ 18903	7.1E-24	1.5E-20	1.5E-20
GO:0042391	regulation of membrane potential	116/ 3120	431/ 18903	2.3E-08	3.7E-05	3.5E-05
GO:0007188	adenylate cyclase-modulating G protein-coupled receptor signaling pathway	69/ 3120	248/ 18903	4.6E-06	5.7E-03	5.5E-03

Supplementary Table S3 Smell-associated genes with significantly regulated transcript levels and a fold-change higher than 1.4 in SSI⁺ IgG⁺ subjects compared to the three adjustment groups.

Abbrev.	Full name	Log_2 fold-change	*p*-value	NCBI ID
OR6C4	Homo sapiens olfactory receptor family 6 subfamily C member 4 (OR6C4). transcript variant 1. mRNA	-0.74	1.21E-04	1883684786
OMP	Homo sapiens olfactory marker protein (OMP). mRNA	-0.56	1.86E-03	5453827
OR2T1	Homo sapiens olfactory receptor family 2 subfamily T member 1 (OR2T1). mRNA	1.40	7.56E-12	1954668725
OR52E8	Homo sapiens olfactory receptor family 52 subfamily E member 8 (OR52E8). mRNA	1.44	1.67E-09	1993776373
clone ht2	H.sapiens mRNA for putative olfactory receptor (clone ht2)	1.64	6.49E-09	2792015
OR10G9	Homo sapiens olfactory receptor family 10 subfamily G member 9 (OR10G9). mRNA	1.48	2,96E-08	50080192
OR6C1	Homo sapiens olfactory receptor family 6 subfamily C member 1 (OR6C1). mRNA	1.20	4.05E-08	1955349240
OR1N2	Homo sapiens olfactory receptor family 1 subfamily N member 2 (OR1N2). mRNA	1.66	5.57E-08	1510189943
OR8B12	Homo sapiens olfactory receptor family 8 subfamily B member 12 (OR8B12). mRNA	1.57	1.18E-07	52353349
OR1L3	Homo sapiens olfactory receptor family 1 subfamily L member 3 (OR1L3). mRNA	1.52	1.74E-07	52546684
OR52E6	Homo sapiens olfactory receptor family 52 subfamily E member 6 (OR52E6). mRNA	1.74	2.56E-07	1653961673
OR5P2	Homo sapiens olfactory receptor family 5 subfamily P member 2 (OR5P2). mRNA	1.32	2.62E-07	23592221
OR4K15	Homo sapiens olfactory receptor family 4 subfamily K member 15 (OR4K15). mRNA	1.40	3.46E-07	1934150438
OR2C1	Homo sapiens olfactory receptor family 2 subfamily C member 1 (OR2C1). mRNA	1.37	3.57E-07	1653961356
OR51A2	Homo sapiens olfactory receptor family 51 subfamily A member 2 (OR51A2). mRNA	1.47	5.13E-07	52317145
OR6M1	Homo sapiens olfactory receptor family 6 subfamily M member 1 (OR6M1). mRNA	1.17	1.15E-06	52693922
OR5M10	Homo sapiens olfactory receptor family 5 subfamily M member 10 (OR5M10). mRNA	0.99	1.23E-06	52317260
OR2B6	Homo sapiens olfactory receptor family 2 subfamily B member 6 (OR2B6). mRNA	0.91	1.96E-06	45504385
OR14K1	Homo sapiens olfactory receptor family 14 subfamily K member 1 (OR14K1). mRNA	1.32	3.54E-06	118766342
OR5K4	Homo sapiens olfactory receptor family 5 subfamily K member 4 (OR5K4). mRNA	1.33	3.58E-06	53933286
OR10V1	Homo sapiens olfactory receptor family 10 subfamily V member 1 (OR10V1). mRNA	1.29	5.01E-06	52693926
OR11A1	Homo sapiens olfactory receptor family 11 subfamily A member 1 (OR11A1). mRNA	1.04	9.45E-06	1877233849
OR56A3	Homo sapiens olfactory receptor family 56 subfamily A member 3 (OR56A3). mRNA	1.46	1.60E-05	1885896390

Supplementary Table S3 Smell associated genes with significantly regulated transcript levels and a fold-change higher than 1.4 in SSI+ IgG+ participants.				
OR4C6	Homo sapiens olfactory receptor family 4 subfamily C member 6 (OR4C6). mRNA	1.46	1.62E-05	1885895917
OR56B1	Homo sapiens olfactory receptor family 56 subfamily B member 1 (OR56B1). mRNA	1.51	1.70E-05	1653962322
OR5M9	Homo sapiens olfactory receptor family 5 subfamily M member 9 (OR5M9). mRNA	0.93	2.47E-05	52317127
OR2D3	Homo sapiens olfactory receptor family 2 subfamily D member 3 (OR2D3). mRNA	0.90	2.64E-05	52317193
OR13C2	Homo sapiens olfactory receptor family 13 subfamily C member 2 (OR13C2). mRNA	1.30	3.88E-05	52317179
OR4F29	Homo sapiens olfactory receptor family 4 subfamily F member 29 (OR4F29). mRNA	1.40	3.96E-05	119943151
OR6K3	Homo sapiens olfactory receptor family 6 subfamily K member 3 (OR6K3). mRNA	1.00	4.11E-05	1885898524
OR6W1	Homo sapiens olfactory receptor. family 6. subfamily W. member 1 pseudogene. mRNA (cDNA clone IMAGE:7262197)	0.63	4.35E-05	47479535
OR52E4	Homo sapiens olfactory receptor family 52 subfamily E member 4 (OR52E4). mRNA	0.89	4.41E-05	1885896105
OR2T12	Homo sapiens olfactory receptor family 2 subfamily T member 12 (OR2T12). transcript variant 1. mRNA	1.06	4.48E-05	1885895712
OR6C3	Homo sapiens olfactory receptor family 6 subfamily C member 3 (OR6C3). transcript variant 1. mRNA	1.13	6.92E-05	1934804125
OR7A5	Homo sapiens olfactory receptor. family 7. subfamily A. member 5. mRNA (cDNA clone MGC:132469 IMAGE:8143812). complete cds	1.10	8.11E-05	85397694
OR7C1	Homo sapiens olfactory receptor family 7 subfamily C member 1 (OR7C1). transcript variant 2. mRNA	0.94	9.14E-05	39812354
OR8D2	Homo sapiens olfactory receptor family 8 subfamily D member 2 (OR8D2). mRNA	0.83	9.68E-05	50897291
OR6C76	Homo sapiens olfactory receptor family 6 subfamily C member 76 (OR6C76). transcript variant 1. mRNA	1.10	9.96E-05	52421785
OR3A1	Homo sapiens olfactory receptor family 3 subfamily A member 1 (OR3A1). mRNA	0.94	1.04E-04	1886308703
OR52B4	Homo sapiens olfactory receptor family 52 subfamily B member 4 (OR52B4). transcript variant 1. coding. mRNA	1.20	1.33E-04	284055262
OR1D2	Homo sapiens olfactory receptor family 1 subfamily D member 2 (OR1D2). transcript variant 1. mRNA	0.79	1.36E-04	1886308713
OR4F16	PREDICTED: Homo sapiens olfactory receptor. family 4. subfamily F. member 16 (OR4F16). mRNA	1.05	1.40E-04	113416854
OR51F1	Homo sapiens olfactory receptor family 51 subfamily F member 1 (OR51F1). mRNA	1.48	1.60E-04	1546946170
OR1A2	Homo sapiens olfactory receptor family 1 subfamily A member 2 (OR1A2). mRNA	0.93	1.63E-04	1933067923
OR4X2	Homo sapiens olfactory receptor family 4 subfamily X member 2 (OR4X2). mRNA	0.72	1.69E-04	52317105
OR4S1	Homo sapiens olfactory receptor family 4 subfamily S member 1 (OR4S1). mRNA	0.77	1.69E-04	52317101
OR5T3	Homo sapiens olfactory receptor family 5 subfamily T member 3 (OR5T3). mRNA	1.05	1.81E-04	1934804072

Supplementary Table S3 Smell associated genes with significantly regulated transcript levels and a fold-change higher than 1.4 in SSI⁺ IgG⁺ participants.				
OR10H2	Homo sapiens olfactory receptor. family 10. subfamily H. member 2. mRNA (cDNA clone MGC:138383 IMAGE:8327646). complete cds	1.31	2.25E-04	85700316
OR2T34	Homo sapiens olfactory receptor family 2 subfamily T member 34 (OR2T34). mRNA	0.84	2.68E-04	49227740
OR51Q1	Homo sapiens olfactory receptor family 51 subfamily Q member 1 (OR51Q1). mRNA	0.67	2.77E-04	284172434
OR4K14	Homo sapiens olfactory receptor family 4 subfamily K member 14 (OR4K14). mRNA	0.92	3.02E-04	1884086693
OR10A5	Homo sapiens olfactory receptor family 10 subfamily A member 5 (OR10A5). mRNA	0.98	3.14E-04	30039687
OR14A16	Homo sapiens olfactory receptor family 14 subfamily A member 16 (OR14A16). mRNA	1.59	3.27E-04	1885010973
OR13C5	Homo sapiens olfactory receptor family 13 subfamily C member 5 (OR13C5). mRNA	0.58	3.29E-04	52218853
OR51V1	Homo sapiens olfactory receptor family 51 subfamily V member 1 (OR51V1). mRNA	0.82	3.50E-04	1993193907
OR4M2	Homo sapiens olfactory receptor family 4 subfamily M member 2 (OR4M2). mRNA	0.83	3.52E-04	284005442
OR4A5	Homo sapiens olfactory receptor family 4 subfamily A member 5 (OR4A5). mRNA	0.92	3.59E-04	284055233
OR2J2	Homo sapiens olfactory receptor family 2 subfamily J member 2 (OR2J2). mRNA	0.76	3.71E-04	1894803088
OR4F17	Homo sapiens olfactory receptor family 4 subfamily F member 17 (OR4F17). mRNA	0.90	3,76E-04	1933067917
OR5AK2	Homo sapiens olfactory receptor family 5 subfamily AK member 2 (OR5AK2). mRNA	0.73	4.51E-04	52693940
OR9K2	Homo sapiens olfactory receptor family 9 subfamily K member 2 (OR9K2). mRNA	1.03	5.27E-04	1889451645
OR13G1	Homo sapiens olfactory receptor family 13 subfamily G member 1 (OR13G1). mRNA	1.20	5.28E-04	1885011206
OR4L1	Homo sapiens olfactory receptor family 4 subfamily L member 1 (OR4L1). mRNA	1.02	5.56E-04	52317258
OR4K2	Homo sapiens olfactory receptor family 4 subfamily K member 2 (OR4K2). mRNA	0.79	5.78E-04	1886833031
OR9Q2	Homo sapiens olfactory receptor family 9 subfamily Q member 2 (OR9Q2). mRNA	0.87	6.27E-04	1894805350
OR5AS1	Homo sapiens olfactory receptor family 5 subfamily AS member 1 (OR5AS1). mRNA	0.82	7.09E-04	1894805373
OR10A3	Homo sapiens olfactory receptor family 10 subfamily A member 3 (OR10A3). mRNA	0.75	7.39E-04	1885895256
OR52J3	Homo sapiens olfactory receptor family 52 subfamily J member 3 (OR52J3). mRNA	0.81	7.79E-04	157041221
OR4F15	Homo sapiens olfactory receptor family 4 subfamily F member 15 (OR4F15). mRNA	0.85	8.31E-04	1885011029
OR1L1	Homo sapiens olfactory receptor family 1 subfamily L member 1 (OR1L1). mRNA	1.12	8.32E-04	256773198
OR7G3	Homo sapiens olfactory receptor family 7 subfamily G member 3 (OR7G3). mRNA	0.87	8.40E-04	50080200
OR8G2	Homo sapiens olfactory receptor. family 8. subfamily G. member 2 (OR8G2). mRNA	1.55	9.09E-04	55925592
OR2T27	Homo sapiens olfactory receptor family 2 subfamily T member 27 (OR2T27). transcript variant 2. mRNA	0.73	1.07E-03	1885895850
OR2G3	Homo sapiens olfactory receptor family 2 subfamily G member 3 (OR2G3). mRNA	0.81	1.08E-03	50054448

Supplementary Table S3 Smell associated genes with significantly regulated transcript levels and a fold-change higher than 1.4 in SSI⁺ IgG⁺ participants.				
OR11H4	Homo sapiens olfactory receptor family 11 subfamily H member 4 (OR11H4). mRNA	0.99	1.09E-03	1886833022
OR52I2	Homo sapiens olfactory receptor family 52 subfamily I member 2 (OR52I2). mRNA	0.74	1.10E-03	2250017162
OR2AG2	Homo sapiens olfactory receptor family 2 subfamily AG member 2 (OR2AG2). transcript variant 1. mRNA	0.90	1.17E-03	1885895214
OR1B1	Homo sapiens olfactory receptor family 1 subfamily B member 1 (OR1B1). transcript variant 1. coding. mRNA	1.01	1.31E-03	2274795572
OR2B2	Homo sapiens olfactory receptor family 2 subfamily B member 2 (OR2B2). mRNA	0.63	1,36E-03	289547695
OR14C36	Homo sapiens olfactory receptor family 14 subfamily C member 36 (OR14C36). mRNA	0.87	1.44E-03	50054469
OR8U8	Homo sapiens olfactory receptor family 8 subfamily U member 8 (OR8U8). mRNA	0.98	1.50E-03	513126878
OR5K1	Homo sapiens olfactory receptor family 5 subfamily K member 1 (OR5K1). mRNA	0.65	1.58E-03	1886308756
OR1S1	Homo sapiens olfactory receptor family 1 subfamily S member 1 (OR1S1). mRNA	1.01	1.75E-03	2272409780
OR5H1	Homo sapiens olfactory receptor family 5 subfamily H member 1 (OR5H1). mRNA	0.79	2.16E-03	1886308743
OR52N2	Homo sapiens olfactory receptor family 52 subfamily N member 2 (OR52N2). mRNA	0.81	2.22E-03	1942116112
OR2T35	Homo sapiens olfactory receptor family 2 subfamily T member 35 (OR2T35). mRNA	0.98	2.30E-03	1885011721
OR52K2	Homo sapiens olfactory receptor family 52 subfamily K member 2 (OR52K2). mRNA	0.72	2.47E-03	284055272
OR12D2	Homo sapiens olfactory receptor. family 12. subfamily D. member 2. mRNA (cDNA clone MGC:126791 IMAGE:8069248). complete cds	0.57	2.50E-03	75517242
OR10R2	Homo sapiens olfactory receptor family 10 subfamily R member 2 (OR10R2). transcript variant 1. mRNA	0.86	2.58E-03	52218841
OR6F1	Homo sapiens olfactory receptor family 6 subfamily F member 1 (OR6F1). mRNA	0.66	2.65E-03	1882652601
OR9G1	Homo sapiens olfactory receptor family 9 subfamily G member 1 (OR9G1). mRNA	0.97	2.88E-03	1880340287
OR6C65	Homo sapiens olfactory receptor family 6 subfamily C member 65 (OR6C65). mRNA	0.79	2.94E-03	53933281
OR4N4	Homo sapiens olfactory receptor family 4 subfamily N member 4 (OR4N4). mRNA	0.60	3.08E-03	1934151739
OR52M1	Homo sapiens olfactory receptor family 52 subfamily M member 1 (OR52M1). mRNA	0.97	3.27E-03	51921274
OR5P2	Homo sapiens olfactory receptor. family 5. subfamily P. member 2. mRNA (cDNA clone MGC:126759 IMAGE:8069216). complete cds	0.62	3.27E-03	75516729
OR8K5	Homo sapiens olfactory receptor family 8 subfamily K member 5 (OR8K5). mRNA	0.76	3.38E-03	145279178
OR52E2	Homo sapiens olfactory receptor family 52 subfamily E member 2 (OR52E2). mRNA	0.77	3.46E-03	284055259
OR13H1	Homo sapiens olfactory receptor family 13 subfamily H member 1 (OR13H1). mRNA	0.53	3.49E-03	52218867
OR4C13	Homo sapiens olfactory receptor family 4 subfamily C member 13 (OR4C13). mRNA	0.77	3.71E-03	284172353

Supplementary Table S3 Smell associated genes with significantly regulated transcript levels and a fold-change higher than 1.4 in SSI⁺ IgG⁺ participants.				
OR1Q1	Homo sapiens olfactory receptor family 1 subfamily Q member 1 (OR1Q1). mRNA	1.19	3.72E-03	50052933
OR2M3	Homo sapiens olfactory receptor family 2 subfamily M member 3 (OR2M3). mRNA	0.80	3.79E-03	1884086696
OR8J1	Homo sapiens olfactory receptor family 8 subfamily J member 1 (OR8J1). mRNA	0.74	3.82E-03	1881766182
OR8G1	Homo sapiens olfactory receptor family 8 subfamily G member 1 (OR8G1). transcript variant 1. coding. mRNA	0.86	3.92E-03	1915575702
OR56A4	PREDICTED: Homo sapiens similar to olfactory receptor. family 56. subfamily A. member 4 (LOC390084). mRNA	0.65	4.07E-03	89033758
OR2T10	Homo sapiens olfactory receptor family 2 subfamily T member 10 (OR2T10). mRNA	0.67	4.18E-03	1890343275
OR6B3	Homo sapiens olfactory receptor family 6 subfamily B member 3 (OR6B3). mRNA	0.65	4.26E-03	1885896587
OR5M1	Homo sapiens olfactory receptor family 5 subfamily M member 1 (OR5M1). mRNA	0.74	4.34E-03	1879298374
OR14J1	Homo sapiens olfactory receptor family 14 subfamily J member 1 (OR14J1). mRNA	0.73	4.42E-03	1877233855
OR1G1	Homo sapiens olfactory receptor family 1 subfamily G member 1 (OR1G1). mRNA	0.95	4.49E-03	11415033
OR2T8	Homo sapiens olfactory receptor family 2 subfamily T member 8 (OR2T8). mRNA	0.73	4.55E-03	1885895404
OR52H1	Homo sapiens olfactory receptor family 52 subfamily H member 1 (OR52H1). mRNA	0.68	4.60E-03	1934804086
OR2A14	Homo sapiens olfactory receptor family 2 subfamily A member 14 (OR2A14). mRNA	0.67	4.79E-03	1878067966
OR4F5	Homo sapiens olfactory receptor family 4 subfamily F member 5 (OR4F5). mRNA	0.55	4.81E-03	1885010411
OR5J2	Homo sapiens olfactory receptor family 5 subfamily J member 2 (OR5J2). mRNA	0.78	4.82E-03	53828704
OR1D4	PREDICTED: Homo sapiens similar to olfactory receptor. family 1. subfamily D. member 4 (LOC653166). mRNA	0.55	4.82E-03	89041143
OR12D3	Homo sapiens olfactory receptor. family 12. subfamily D. member 3. mRNA (cDNA clone MGC:95357 IMAGE:7216896). complete cds	0.82	4.98E-03	46575729
OR1D5	PREDICTED: Homo sapiens olfactory receptor. family 1. subfamily D. member 5 (OR1D5). mRNA	0.78	5.05E-03	89041145
OR2AG1	Homo sapiens olfactory receptor family 2 subfamily AG member 1 (OR2AG1). mRNA	0.88	5,77E-03	1894805396
OR1A1	Homo sapiens olfactory receptor family 1 subfamily A member 1 (OR1A1). transcript variant 1. mRNA	0.56	5.96E-03	1886832967
OR4D9	Homo sapiens olfactory receptor family 4 subfamily D member 9 (OR4D9). mRNA	0.79	6.03E-03	1887096792
OR5AN1	Homo sapiens olfactory receptor family 5 subfamily AN member 1 (OR5AN1). mRNA	0.76	6.07E-03	1878067972
OR5D16	Homo sapiens olfactory receptor family 5 subfamily D member 16 (OR5D16). mRNA	0.90	6.24E-03	53828709
OR2M4	Homo sapiens olfactory receptor family 2 subfamily M member 4 (OR2M4). mRNA	0.62	6.56E-03	1884086692
OR10G2	Homo sapiens olfactory receptor family 10 subfamily G member 2 (OR10G2). mRNA	0.93	6.65E-03	612339331

Supplementary Table S3 Smell associated genes with significantly regulated transcript levels and a fold-change higher than 1.4 in SSI+ IgG+ participants.				
OR7E91	Homo sapiens olfactory receptor. family 7. subfamily E. member 91 pseudogene. mRNA (cDNA clone IMAGE:3996998)	0.75	6.79E-03	15680090
OR10Z1	Homo sapiens olfactory receptor family 10 subfamily Z member 1 (OR10Z1). mRNA	0.58	7.27E-03	1883543198
OR1C1	Homo sapiens olfactory receptor family 1 subfamily C member 1 (OR1C1). mRNA	0.51	7.88E-03	1884086651
OR2F2	Homo sapiens olfactory receptor family 2 subfamily F member 2 (OR2F2). mRNA	0.66	7.89E-03	52317195
OR10H2	Homo sapiens olfactory receptor family 10 subfamily H member 2 (OR10H2). mRNA	1.16	8.34E-03	154816189
OR2A5	Homo sapiens olfactory receptor family 2 subfamily A member 5 (OR2A5). mRNA	0.57	8.76E-03	1878067958
OR6B2	Homo sapiens olfactory receptor. family 6. subfamily B. member 2. mRNA (cDNA clone IMAGE:6178666). with apparent retained intron	0.78	9.15E-03	41388890
OR2F1	Homo sapiens olfactory receptor family 2 subfamily F member 1 (OR2F1). mRNA	0.69	9.86E-03	1887789615
OR2H2	Homo sapiens olfactory receptor. family 2. subfamily H. member 2. mRNA (cDNA clone MGC:95431 IMAGE:7217006). complete cds	0.80	9.94E-03	46575691
OR1D5	Homo sapiens olfactory receptor family 1 subfamily D member 5 (OR1D5). mRNA	0.69	1.00E-02	7657422
OR10J3	Homo sapiens olfactory receptor family 10 subfamily J member 3 (OR10J3). mRNA	0.67	1,01E-02	52353950
OR51S1	Homo sapiens olfactory receptor family 51 subfamily S member 1 (OR51S1). mRNA	1.02	1.01E-02	52317153
OR10J5	Homo sapiens olfactory receptor family 10 subfamily J member 5 (OR10J5). mRNA	0.55	1.09E-02	52218837
ORP	Homo sapiens mRNA for olfactory receptor protein. partial	0.62	1.11E-02	2370144
OR10S1	Homo sapiens olfactory receptor family 10 subfamily S member 1 (OR10S1). mRNA	0.59	1.15E-02	2056392276
OR52R1	Homo sapiens olfactory receptor family 52 subfamily R member 1 (OR52R1). mRNA	0.57	1.20E-02	281182746
OR10H4	Homo sapiens olfactory receptor family 10 subfamily H member 4 (OR10H4). mRNA	0.97	1.28E-02	52218833
OR7G1	Homo sapiens olfactory receptor family 7 subfamily G member 1 (OR7G1). mRNA	0.70	1.29E-02	308737006
OR10K2	Homo sapiens olfactory receptor family 10 subfamily K member 2 (OR10K2). mRNA	0.63	1.43E-02	1885010708
OR6A2	Homo sapiens olfactory receptor. family 6. subfamily A. member 2. mRNA (cDNA clone MGC:126538 IMAGE:8068995). complete cds	0.68	1.53E-02	75516943
OR7C2	Homo sapiens olfactory receptor family 7 subfamily C member 2 (OR7C2). mRNA	0.54	1.54E-02	13624324
OR52B6	Homo sapiens olfactory receptor family 52 subfamily B member 6 (OR52B6). mRNA	1.09	1.65E-02	148229438
OR6Y1	Homo sapiens olfactory receptor family 6 subfamily Y member 1 (OR6Y1). transcript variant 1. mRNA	0.75	1.88E-02	1885010485
OR139	PREDICTED: Homo sapiens similar to olfactory receptor 139 (LOC642966). mRNA	0.73	1.92E-02	89041147

Supplementary Table S3 Smell associated genes with significantly regulated transcript levels and a fold-change higher than 1.4 in SSI⁺ IgG⁺ participants.				
OR52I1	Homo sapiens olfactory receptor family 52 subfamily I member 1 (OR52I1). mRNA	0.54	2.19E-02	52353333
OR5B3	Homo sapiens olfactory receptor family 5 subfamily B member 3 (OR5B3). mRNA	0.72	2.23E-02	1885011173
OR4F6	Homo sapiens olfactory receptor family 4 subfamily F member 6 (OR4F6). mRNA	0.73	2.27E-02	1885011741
OR8U1	Homo sapiens olfactory receptor family 8 subfamily U member 1 (OR8U1). mRNA	0.52	2.29E-02	52353303
OR10H5	Homo sapiens olfactory receptor family 10 subfamily H member 5 (OR10H5). mRNA	0.61	2.37E-02	1886308693
OR2Z1	Homo sapiens olfactory receptor family 2 subfamily Z member 1 (OR2Z1). mRNA	0.50	2.45E-02	1954668686
OR4N5	Homo sapiens olfactory receptor family 4 subfamily N member 5 (OR4N5). mRNA	0.53	2.51E-02	1885011207
OR5AR1	Homo sapiens olfactory receptor family 5 subfamily AR member 1 (OR5AR1). mRNA	0.54	2,52E-02	52317103
OR2T3	Homo sapiens olfactory receptor family 2 subfamily T member 3 (OR2T3). mRNA	0.52	2.59E-02	53828741
OR4A47	Homo sapiens olfactory receptor family 4 subfamily A member 47 (OR4A47). mRNA	0.65	2.68E-02	156119606
OR4D6	Homo sapiens olfactory receptor family 4 subfamily D member 6 (OR4D6). mRNA	0.68	2.85E-02	52317244
OR2S2	Homo sapiens olfactory receptor family 2 subfamily S member 2 (OR2S2). mRNA	0.71	3.07E-02	167830487
OR2T11	Homo sapiens olfactory receptor family 2 subfamily T member 11 (OR2T11). mRNA	0.54	3.22E-02	1885895875
OR2T33	Homo sapiens olfactory receptor family 2 subfamily T member 33 (OR2T33). mRNA	0.56	3.30E-02	1883542064
OR4A15	Homo sapiens olfactory receptor family 4 subfamily A member 15 (OR4A15). mRNA	0.59	3,36E-02	2054205004
OR10H1	Homo sapiens olfactory receptor family 10 subfamily H member 1 (OR10H1). mRNA	1.04	3.46E-02	1886308745
OR10G7	Homo sapiens olfactory receptor family 10 subfamily G member 7 (OR10G7). mRNA	0.69	3.95E-02	1885894816
OR1L8	Homo sapiens olfactory receptor family 1 subfamily L member 8 (OR1L8). mRNA	0.59	4.63E-02	1953526466
OR4D1	Homo sapiens olfactory receptor family 4 subfamily D member 1 (OR4D1). transcript variant 2. mRNA	0.71	4.66E-02	1886833014
OR51E2	Homo sapiens olfactory receptor family 51 subfamily E member 2 (OR51E2). mRNA	0.55	4.93E-02	1653961057

Supplementary Table S4 Taste associated genes with significantly lower transcript levels and a fold-change higher than 1.4 in SSI+ IgG+ subjects compared to the three adjustment groups.

Abbrev.	Description	Log_2 fold-change	*p*-value	NCBI ID
TAS1R1	Homo sapiens taste receptor, type 1, member 1, transcript variant 1, mRNA	1.22	1.36E-07	29294616
TAS2R1	Homo sapiens taste receptor, type 2, member 1, mRNA (cDNA clone MGC:126778 IMAGE:8069235), complete cds	1.22	1.44E-06	75517854
TAS2R60	Homo sapiens taste 2 receptor member 60, mRNA	1.40	6.52E-05	28973794
TAS1R1	Homo sapiens taste 1 receptor member 1, transcript variant 2, mRNA	0.91	1.50E-04	1519311505
TAS2R20	Homo sapiens taste 2 receptor member 20, mRNA	0.66	2.39E-04	1890284530
TAS2R50	Homo sapiens taste receptor, type 2, member 50, mRNA (cDNA clone MGC:138305 IMAGE:8327568), complete cds	1.02	5.87E-04	85566775
TAS2R3	Homo sapiens taste 2 receptor member 3, mRNA	0.77	1.07E-03	67944636
TAS2R7	Homo sapiens taste receptor, type 2, member 7, mRNA (cDNA clone MGC:121027 IMAGE:7939837), complete cds	0.51	3.67E-03	62740071
TAS2R42	Homo sapiens taste 2 receptor member 42, mRNA	0.68	4.03E-03	612149741
TAS2R41	Homo sapiens taste 2 receptor member 41, mRNA	0.59	1.38E-02	116268092

Supplementary Table S5 Metal perception-associated genes included in the analysis

Abbrev.	Full name	Log_2 fold-change	*p*-value	NCBI ID	Literature
OR1A2	Homo sapiens olfactory receptor family 1 subfamily A member 2 (OR1A2). mRNA	0.93	1.63E-04	1933067923	[59,60]
OR2J2	Homo sapiens olfactory receptor family 2 subfamily J member 2 (OR2J2). mRNA	0.76	3.71E-04	1894803088	[60]
OR5K1	Homo sapiens olfactory receptor family 5 subfamily K member 1 (OR5K1). mRNA	0.65	1.58E-03	1886308756	[23,53–55]
OR2M3	Homo sapiens olfactory receptor family 2 subfamily M member 3 (OR2M3). mRNA	0.80	3.79E-03	1884086696	[21,22]
OR1G1	Homo sapiens olfactory receptor family 1 subfamily G member 1 (OR1G1). mRNA	0.95	4.49E-03	11415033	[61]
MTF1	Homo sapiens metal regulatory transcription factor 1 (MTF1). mRNA	-0.64	4.66E-03	1519314509	[52]
OR1A1	Homo sapiens olfactory receptor family 1 subfamily A member 1 (OR1A1). transcript variant 1. mRNA	0.56	5.96E-03	1886832967	[59]
SLC48A1	Homo sapiens solute carrier family 48 member 1 (SLC48A1). mRNA	-0.45	6.51E-03	1519311386	[81]
SLC11A2	Homo sapiens solute carrier family 11 member 2 (SLC11A2). transcript variant 4. mRNA	0.31	7.32E-02	1677538535	[81]
TFRC	Homo sapiens transferrin receptor (TFRC). transcript variant 1. mRNA	-0.28	9.20E-02	1676317495	[81]
TRPM5	Homo sapiens transient receptor potential cation channel subfamily M member 5 (TRPM5). mRNA	-0.31	9.30E-02	2067662279	[21]
OR52D1	Homo sapiens olfactory receptor family 52 subfamily D member 1 (OR52D1). mRNA	0.32	1.14E-01	284172436	[61]
TRPA1	Homo sapiens transient receptor potential cation channel subfamily A member 1 (TRPA1). mRNA	-0.22	1.19E-01	1519316375	[27]
OR56A4	Homo sapiens olfactory receptor family 56 subfamily A member 4 (OR56A4). mRNA	0.55	1.41E-01	1880631221	[82,83]
LCN2	Homo sapiens lipocalin 2 (LCN2). mRNA	-0.21	1.93E-01	1519312321	[81]
SLC40A1	Homo sapiens solute carrier family 40 member 1 (SLC40A1). mRNA	0.26	2.24E-01	1780222516	[81]

Supplementary Table S5 Metal perception-associated genes included in the analysis					
IREB2	Homo sapiens iron responsive element binding protein 2 (IREB2). transcript variant 1. mRNA	-0.19	3.16E-01	1519312869	[81]
TRPV1	Homo sapiens transient receptor potential cation channel subfamily V member 1 (TRPV1). transcript variant 2. mRNA	0.16	3.21E-01	117306160	[21,27,84]
TAS2R7	Homo sapiens taste 2 receptor member 7 (TAS2R7). mRNA	-0.10	5.57E-01	68160950	[27,50]
TAS1R3	Homo sapiens taste 1 receptor member 3 (TAS1R3). mRNA	-0.08	6.65E-01	1519312880	[27,85]

Supplementary Table S6 Metal associated genes with regulated transcript levels and a fold-change higher than 1.4 in SSI$^+$ IgG$^+$ subjects compared to the three adjustment groups.

**Abbrev.**	**Full name**	**Log$_2$ fold-change**	***p*-value**	**NCBI ID**
MTF1	Homo sapiens metal regulatory transcription factor 1 (MTF1), mRNA	- 0.64	4.66E-03	1519314509
OR1A2	Homo sapiens olfactory receptor family 1 subfamily A member 2 (OR1A2), mRNA	0.93	1.63E-04	1933067923
OR2J2	Homo sapiens olfactory receptor family 2 subfamily J member 2 (OR2J2), mRNA	0.76	3.71E-04	1894803088
OR5K1	Homo sapiens olfactory receptor family 5 subfamily K member 1 (OR5K1), mRNA	0.65	1.58E-03	1886308756
OR2M3	Homo sapiens olfactory receptor family 2 subfamily M member 3 (OR2M3), mRNA	0.80	3.79E-03	1884086696
OR1G1	Homo sapiens olfactory receptor family 1 subfamily G member 1 (OR1G1), mRNA	0.95	4.49E-03	11415033
OR1A1	Homo sapiens olfactory receptor family 1 subfamily A member 1 (OR1A1), transcript variant 1, mRNA	0.56	5.96E-03	1886832967
